# The evolution of antibiotic resistance in a structured host population

**DOI:** 10.1098/rsif.2018.0040

**Published:** 2018-06-20

**Authors:** François Blanquart, Sonja Lehtinen, Marc Lipsitch, Christophe Fraser

**Affiliations:** 1 Centre for Interdisciplinary Research in Biology (CIRB), Collège de France, CNRS, INSERM, PSL Research University, Paris, France; 2 IAME, UMR 1137, INSERM, Université Paris Diderot, Site Xavier Bichat, Paris, France; 3 Big Data Institute, Nuffield Department of Medicine, University of Oxford, Oxford, UK; 4 Department of Infectious Disease Epidemiology, Imperial College London, London, UK; 5 Center for Communicable Disease Dynamics, Harvard T. H. Chan School of Public Health, Boston, MA, USA; 6 Department of Epidemiology, Harvard T. H. Chan School of Public Health, Boston, MA, USA; 7 Department of Immunology and Infectious Diseases, Harvard T. H. Chan School of Public Health, Boston, MA, USA

**Keywords:** drug resistance, antimicrobial resistance, mathematical model, adaptation, heterogeneous environment, community

## Abstract

The evolution of antibiotic resistance in opportunistic pathogens such as *Streptococcus pneumoniae*, *Escherichia coli* or *Staphylococcus aureus* is a major public health problem, as infection with resistant strains leads to prolonged hospital stay and increased risk of death. Here, we develop a new model of the evolution of antibiotic resistance in a commensal bacterial population adapting to a heterogeneous host population composed of untreated and treated hosts, and structured in different host classes with different antibiotic use. Examples of host classes include age groups and geographic locations. Explicitly modelling the antibiotic treatment reveals that the emergence of a resistant strain is favoured by more frequent but shorter antibiotic courses, and by higher transmission rates. In addition, in a structured host population, localized transmission in host classes promotes both local adaptation of the bacterial population and the global maintenance of coexistence between sensitive and resistant strains. When transmission rates are heterogeneous across host classes, resistant strains evolve more readily in core groups of transmission. These findings have implications for the better management of antibiotic resistance: reducing the rate at which individuals receive antibiotics is more effective to reduce resistance than reducing the duration of treatment. Reducing the rate of treatment in a targeted class of the host population allows greater reduction in resistance, but determining which class to target is difficult in practice.

## 1. Introduction

The evolution of antimicrobial resistance in bacteria is an important public health problem [1], as infection with resistant strains leads to prolonged hospital stay and increased risk of death [2–6]. Many infections are caused by bacterial species that are mostly commensal, but also sometimes opportunistic pathogens. For example, *Streptococcus pneumoniae*, carried mainly by children [7], causes otitis, meningitis, bacteraemia and pneumonia and accounted before vaccine introduction for approximately 800 000 deaths per year [8]. Multiple genotypes resistant to antibiotics have emerged worldwide in past years in these mainly commensal species, and the frequency of most types of resistance has stabilized at an intermediate level. In *S. pneumoniae*, resistance is associated with globally distributed clones [9–12], has remained stable in the USA and in Europe over the last 15–20 years [13–15] and correlates with levels of antibiotic use across countries [16], suggesting that antibiotics exert strong selection favouring resistant strains.

Although it may seem intuitive that resistance has evolved because of antibiotic use, the stable equilibrium of resistance at an intermediate level, observed for several types of resistance in several species, is surprising. Models show that depending on the balance between the rate of treatment and the cost of resistance, the resistant strain is either unable to invade, or goes to fixation in the population [17]. The inability of simple models to reproduce coexistence was noted in the specific contexts of *S. pneumoniae* [15,18,19] and *Staphylococcus aureus* [20] and is an instance of the more general problem of the maintenance of genetic diversity in the face of selection in evolutionary biology [21].

Recently, it has been proposed that coexistence of sensitive and resistant strains may be maintained because resistance is favoured in strains with longer duration of carriage, creating a genetic association between duration of carriage and resistance: accordingly, in *S. pneumoniae*, resistance is associated with capsular types with longer carriage [22]. Here, we explore the alternative (and non-exclusive) hypothesis that the evolutionary force maintaining the stable frequency of resistance is adaptation to a structured host population. Indeed, antibiotic use and frequency of resistance strongly correlate across European countries in several species [16]; resistance is more frequent in younger age classes that use antibiotics more frequently [23]; simulations of multiple resistances in *S. aureus* showed that population structure enhances coexistence [20]. In spite of the potential importance of population structure for the evolution of resistance, few epidemiological models of the evolution of resistance in commensal bacteria in a structured population exist [24,25]. There is a long tradition of theoretical population genetics studies of adaptation and maintenance of diversity in heterogeneous environments [26–28]. But these models do not account for epidemiological variability: bacteria evolving in a structured host population experience an environment variable across hosts but also over time, as antibiotic treatment is transient, and the epidemiological dynamics of the host population additionally create variability in the density of hosts available to the bacterial population.

We build an epidemiological–evolutionary model to study the evolution of antibiotic resistance in bacteria colonizing a host population structured into different classes that vary in their rate of antibiotic treatment. The model considers the evolution of resistance in bacterial species with a mainly *commensal* lifestyle, whereby the population experiences antibiotic treatment at a low rate and largely independently of colonization by the focal species. This situation is different from an obligate bacterial pathogen, where asymptomatic carriage is rare and antibiotic selection arises principally due to infection itself [29]. We begin by introducing the model describing the epidemiology and evolution of an antibiotic-sensitive and an antibiotic-resistance bacterial strain. We then define the *invasion fitness* of a strain, which is the exponential growth rate of that strain when it is rare and invading a resident population. We derive several analytical approximations for invasion fitness of a sensitive and a resistant strain, and examine these expressions to gain insights into the factors favouring the evolution of resistant strains, and the maintenance of coexistence of resistant and sensitive strains. Lastly, we use the model to predict the impact of public health measures to reduce antibiotic resistance.

## 2. The model

### 2.1. An epidemiological model for the evolution of resistance in a structured population

We model the evolution of antibiotic resistance in a host population divided into several classes using a compartmental ordinary differential equation (ODE) model (variables and parameters are described in table 1). Hosts may be uncolonized (the variable *X* denotes the density of such hosts), colonized by an antibiotic sensitive strain (variable *S*) or colonized by an antibiotic resistant strain (variable *R*). Uncolonized individuals are often called ‘susceptibles’ in infectious disease epidemiology, but we do not use this terminology to avoid confusion with antibiotic sensitivity. Resistance is associated with clones or plasmids circulating in the population (‘transmitted’ or ‘primary’ resistance), and rarely evolves de novo within a treated host (‘acquired’ resistance). Additionally, hosts may be untreated or treated by antibiotics, denoted with a superscript ‘*U*’ or ‘*T*’. Different classes of hosts are denoted with a subscript *i*. Classes represent properties of the host population, for example, age, or country, and in the most general model, determine the rates of treatment, clearance and transmission. We follow the density of hosts of each type, described by the variables 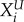 and 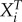, 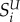 and 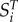, 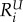 and 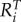, for *i* in [1; *n*], where *n* is the number of classes. 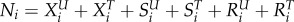 denotes the size of class *i*. We assume transitions of hosts between classes occur on a slow timescale relative to the other timescales considered in this paper, and may be neglected, so the size of classes *N_i_* is constant in time. Four different events may occur and change the value of these variables.

#### 2.1.1. Treatment start

An untreated *uncolonized* host of class *i* goes on treatment at rate *τ_i_*, while an untreated *colonized* host goes on treatment at rate 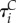. Typically, 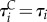 when hosts use antibiotics independently of colonization with the focal species: in that case, the focal species experiences ‘bystander’ or ‘collateral’ selection as a result of antibiotic use caused by infection with other bacterial (or viral) species. When 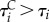, the focal species occasionally causes infections and experiences direct antibiotic selection for treatment of these infections. We will assume throughout that the rate of treatment is smaller than epidemiological events of transmission and clearance (see below).

#### 2.1.2. Treatment cessation

A treated host of class *i* goes back to the ‘untreated’ category with rate *ω_i_*. Therefore, 1/*ω_i_* is the expected duration of an antibiotic course.

#### 2.1.3. Clearance

A colonized host of class *i* clears the bacteria at rates *u_i_*_,*S*_ and *u_i_*_,*R*_ for an *untreated* host colonized by the sensitive and resistant strains, respectively; and *u_i_*_,*S*_ + *a_S_* and *u_i_*_,*R*_ + *a_R_* for a *treated* host colonized by the sensitive and resistant strains. The coefficients *a* represent the action of antibiotics increasing the rate of clearance. *a_R_* is 0 for a perfectly resistant strain. *a_S_* tends to infinity for a fully sensitive strain instantaneously cleared by the antibiotic—in that case, the compartment of sensitive-colonized treated 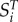 is negligible as the treated host instantaneously become uncolonized.

#### 2.1.4. Transmission

An uncolonized host of class *j* gets colonized by a host of class *i*, at a rate 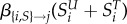 for colonization by a sensitive strain, and 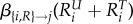 for colonization by a resistant strain. We do not consider dual colonization in our model, as it introduces many complexities in model choice not strictly relevant to the topic of our model analysis [18]. Transmissibility of either strain does not depend on the treatment status of the colonized host.

The following ODE describe the dynamics of the system:
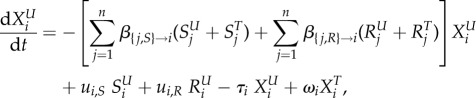

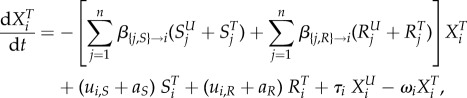

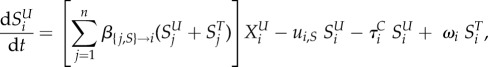

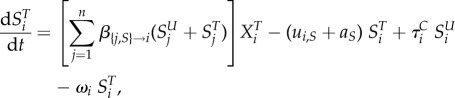

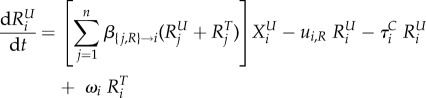

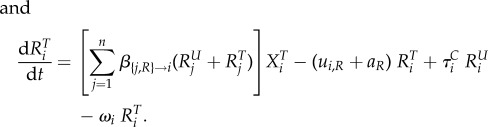


The model is similar in its structure to a classical model of the evolution of antibiotic resistance in the community, with an explicit description of antibiotic treatment [30]. Here, we additionally consider a structured host population and allow for partial sensitivity and resistance.

We assume the total population size is constant, set to 1 without loss of generality. We reduce the number of equations using two sets of constraints. First, hosts do not change classes over the timescales relevant to our model, such that the fraction of hosts in class *i* remains constant, equal to *N_i_*. Second, we assume the fraction of treated hosts in each class is always at its equilibrium given by 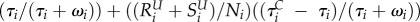. These relationships enable us to consider only the dynamics of the colonized individuals, from which the dynamics of uncolonized individuals follow.

**Table 1.** Table of variables and parameters.

**variables**	
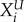	density of uncolonized, untreated hosts in class *i*
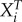	density of uncolonized, treated hosts in class *i*
*X^U^*	total density of uncolonized, untreated hosts, equal to 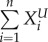
*X^T^*	total density of uncolonized, treated hosts, equal to 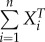
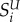	density of untreated hosts colonized by a sensitive bacterial strain in class *i*
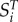	density of treated hosts colonized by a sensitive bacterial strain in class *i*
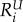	density of untreated hosts colonized by a resistant bacterial strain in class *i*
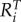	density of treated hosts colonized by a resistant bacterial strain in class *i*
*p*	frequency of resistance, equal to 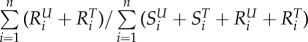
**parameters**	
*N_i_*	total host density in class *i*, assumed to be constant
*n*	number of host classes. Host classes are numbered from 1 to *n*
*β*_{_*_i_*_,*S*}→_*_j_*	rate of transmission of the *sensitive* strain, from a colonized individual of class *i* to an uncolonized individual of class *j*, month^−1^
*β*_{_*_i_*_,*R*}→_*_j_*	rate of transmission of the *resistant* strain, from a colonized individual of class *i* to an uncolonized individual of class *j*, month^−1^
*ɛ*	proportion of inter-class transmission, used in some versions of the model
*c*	the transmission cost of resistance
*u_i_*_,*S*_	rate of natural clearance of the *sensitive* strain in host class *i*, month^−1^
*u_i_*_,*R*_	rate of natural clearance of the *resistant* strain in host class *i*, month^−1^
*a_S_*	rate of antibiotic clearance for a *sensitive* strain, month^−1^
*a_R_*	rate of antibiotic clearance for a *resistant* strain, month^−1^
*τ_i_*	rate of antibiotic treatment in class *i* for an uncolonized host, month^−1^
*τ*	mean rate of antibiotic treatment in the uncolonized host population, 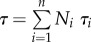
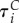	rate of antibiotic treatment in class *i* for a colonized host, month^−1^
*τ ^C^*	mean rate of antibiotic treatment in the colonized host population, 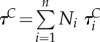
*f ^C^*	multiplicative factor equal to the treatment rate of colonized hosts over that in uncolonized hosts, 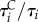 , assumed to be constant across classes
*ω_i_*	rate of treatment cessation per month. 1/*ω_i_* is the average antibiotic course duration

### 2.2. Invasion fitness predicts the coexistence of a sensitive and a resistant strain

Our goal is to study the factors favouring the emergence of sensitive and resistant strains. To this end, we define the *invasion fitness*. Invasion fitness is the exponential growth rate of a focal strain (the ‘mutant’, which can be more sensitive or more resistant than the resident), when it is still rare and invading a population of the resident at equilibrium. The mutant strain increases in frequency when rare when invasion fitness is positive [31]. Invasion fitness is the relevant quantity to describe the dynamics of different bacterial strains in an endemic pathogen, and is distinct from the basic reproduction number *R*_0_ which describes the initial dynamics of a pathogen in a fully uncolonized host population. Invasion fitness is specifically the dominant eigenvalue of the matrix describing the linearized dynamics of the rare mutant, denoted *λ_S_* and *λ_R_* for the sensitive and resistant strains (electronic supplementary material).

The invasion fitnesses are used to perform a stability analysis of the equilibria. The trivial equilibrium **0** where the pathogen is absent is assumed to be unstable. For a class of models similar to ours, if the pathogen population grows when rare and declines when large enough, there exists a positive compact global attractor (theorem 3.18 in [32]). Conjecturing this result remains true for our slightly different system, this global attractor can be either one of the two monomorphic equilibria^1^ (with only the *S* or *R* strain), if one of them is stable, or a global attractor where the two strains coexist. From these considerations, three situations may occur depending on the sign of *λ_S_* and *λ_R_*:

(i) *λ_S_* < 0 and *λ_R_* > 0, the resistant strain can invade the sensitive but the reverse is not true: the resistant strain takes over the population.
(ii) *λ_S_* > 0 and *λ_R_* < 0, the sensitive strain can invade the resistant but the reverse is not true: the sensitive strain takes over the population.
(iii) *λ_S_* > 0 and *λ_R_* > 0, each strain can invade the other. Coexistence of the two strains is possible.

When the sensitive and resistant strains coexist (case (iii)), the invasion fitnesses not only predict growth of a mutant strain when invading the resistant strain, but also correlate with the resulting equilibrium frequency of resistance. Specifically, defining *p* as the frequency of resistance, *p*/(1 − *p*) correlates well with *λ_R_*/*λ_S_* across a range of randomly chosen parameter values (*R*^2^ = 0.75 on log-scale, electronic supplementary material, figure S1). Thus, the dependence of the invasion fitnesses on the parameters reveals the factors favouring the sensitive or the resistant strain when the two strains coexist.

In the following, we derive expressions for *λ_S_* and *λ_R_* and examine the factors favouring resistance, sensitivity and coexistence of the two strains.

## 3. Results

### 3.1. Analytical expressions for invasion fitness

The main analytical result is a set of approximations for the invasion fitness of a mutant strain (denoted by a subscript '*M*') invading a resident strain (denoted by a subscript '*WT*' for wild-type), that can interchangeably denote the resistant ('*R*') or sensitive ('*S*') strain. We assume throughout that treatment rates (*τ_i_* and 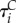) are smaller than rates governing epidemiological events (transmission, clearance), and the results are first-order Taylor approximations in the treatment rates. We derive approximations for *λ_M_* in three scenarios for transmission. In the first scenario, ‘no inter-class transmission’, transmission only occurs within classes but not between classes. In mathematical terms, *β*_{_*_i_*_,*R*}→_*_j_* = 0 and *β*_{_*_i_*_,*S*}→_*_j_* = 0 when *i* ≠ *j*. In the second scenario, the rates of inter-class transmission are small but non-zero. In the third scenario, ‘full inter-class transmission’, transmission between and within classes occurs at the same rate, that is, *β*_{_*_i_*_,*R*}→_*_j_* = *β_R_* and *β*_{_*_i_*_,*S*}→_*_j_* = *β_S_* for all *i*, *j*. The transmission cost of resistance is 1 − *β_R_*/*β_S_*. In that third scenario, to obtain simple results, we also assume the clearance rates *u_i_*_,*S*_ and *u_i_*_,*R*_ are the same in all classes (equal to *u_S_* and *u_R_*). The invasion fitnesses in the three scenarios are the following:
3.1
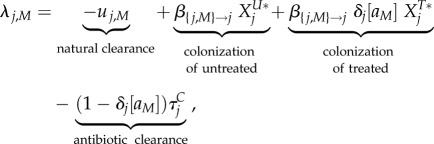

3.2
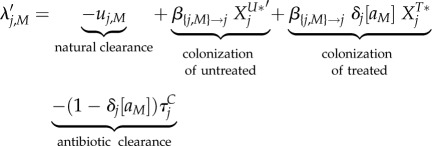
and
3.3
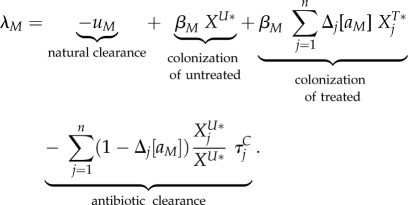


Four phenomena determine the invasion fitness. The first term is negative and represents natural clearance of the bacteria by the host. The second and third terms are positive and represent growth of the mutant by colonization of untreated and treated uncolonized hosts denoted by 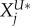, 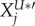, X^U^*, and 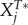. These are the densities of the equilibrium resident population, denoted by a star. The fourth term is negative and represents antibiotic clearance.

The invasion fitness takes a different form in the different scenarios. When there is no inter-class transmission (equation (3.1)), each eigenvalue of the matrix describing the linearized dynamics of the rare mutant represents growth in each of the classes. For a mutant genotype *M* invading an equilibrium population of a resident genotype, and with maximal initial growth in class *j*, the invasion fitness is given by considering the initial growth in class *j*. Adding a small rate of inter-class transmission (equation (3.2)) does not change the form of the invasion fitness to the first order in this small rate, but reduces the density of uncolonized untreated hosts from 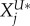 to 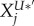 (electronic supplementary material). Under full inter-class transmission (equation (3.3)), the population effectively behaves similarly to a single well-mixed population where the densities of uncolonized hosts and the antibiotic clearance are averaged over the host population (X^U^* is the total density of uncolonized hosts at equilibrium).

An important quantity in the invasion fitness is 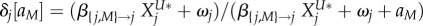 (and the analogous 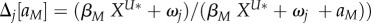. This quantity modulates both the capacity of the mutant to invade the compartment of treated hosts 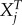 and the impact of antibiotic treatment on the mutant. It is 1 when *a_M_* = 0 (mutant with full resistance, unaffected by antibiotics) and declines to 0 as *a_M_* → ∞ (mutant with full sensitivity, instantaneously cleared by antibiotics). A strain with 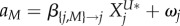 behaves exactly intermediately between a fully resistant and fully sensitive strain, *δ_j_*[*a_M_*] = 1/2.

Equation (3.3) can be completed with the equilibrium expressions for the densities of uncolonized hosts at the resident equilibrium, given in electronic supplementary material.

### 3.2. Interpretation of the invasion fitness for the sensitive and resistant strains

Equations (3.1)–(3.3) give the generic invasion fitness of a mutant invading a resident pathogen population. We can now adapt these equations to the specific case of a resistant (resp. sensitive) strain invading a sensitive (resp. resistant) strain. The main parameter that will be affected by the level of resistance is *a*, with *a_R_* < *a_S_*. We also expect resistance to be costly, such that the resistant strain transmits less (*β_R_* < *β_S_*) and is naturally cleared faster by the host (*u_R_* > *u_S_*). The value of *a* will in particular affect the quantity *δ_j_*[*a_M_*] (and Δ*_j_*[*a_M_*]) and therefore the strategy on which the strain relies for replication. In the extreme case of a perfectly resistant strain and a perfectly sensitive strain (*a_S_* → ∞ and *a_R_* = 0), the invasion fitnesses (for example, in the full inter-class transmission scenario but the expressions would be straightforwardly transposed to the other scenarios) are:
3.4a
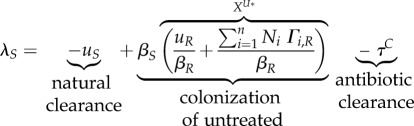

3.4b
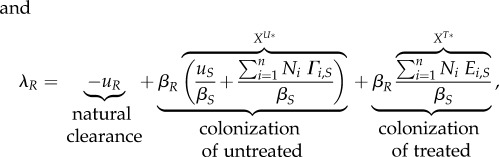
where *Γ_i_*_,*R*_ and *Γ_i_*_,*S*_ are small terms representing the impact of antibiotic treatment on the density of uncolonized untreated hosts and *E_i_*_,*S*_ is a small positive term (electronic supplementary material). This reveals how the sensitive and resistant strains rely on different strategies for replication. The perfectly sensitive strain replicates in untreated hosts, but its growth is hindered by antibiotic prescribed at a rate *τ^C^*. However, it may typically replicate faster than the resistant strain 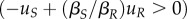 because of the cost of resistance. The perfectly resistant strain is unaffected by treatment and additionally benefits from colonizing the treated uncolonized hosts who form an ecological niche for that strain, as already shown in a well-mixed population model [30].

**Figure 1.**
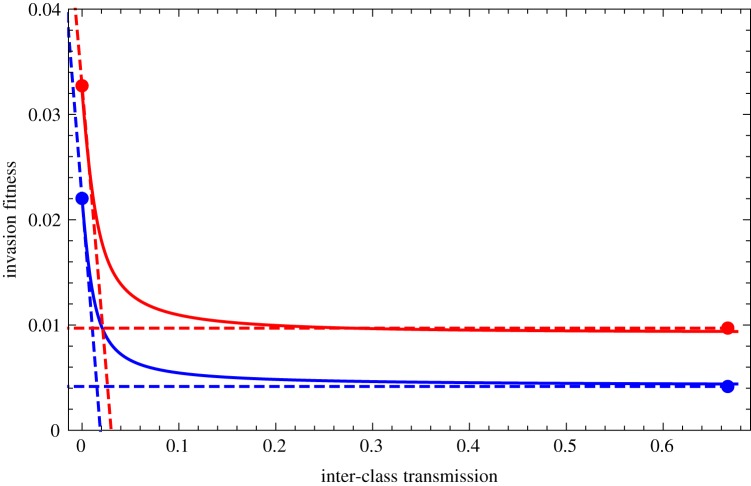
The invasion fitness *λ_S_* (blue) and *λ_R_* (red) are shown as a function of inter-class transmission. The plain line is the simulation, the bullets are the ‘no inter-class transmission’ and ‘full inter-class transmission’ approximations (equations (3.1) and (3.3)), the horizontal dashed lines shows again the full inter-class transmission approximation (equation (3.3)) and the declining dashed lines the Taylor series analysis around 0 inter-class transmission (equation (3.2)). We model three classes of equal sizes *N*_1_ = *N*_2_ = *N*_3_ = 1/3. The baseline parameters are clearance rates *u_S_* = *u_R_* = 1.1 month^−1^; treatment cessation *ω* = 4 month^−1^; baseline transmission rate *β* = 10 month^−1^, cost of resistance *c* = 0.04; treatment varies across three classes, with rates *τ*_1_ = 0.02, *τ*_2_ = 0.035, *τ*_3_ = 0.05 month^−1^ and treatment is 10% higher in the colonized individuals, 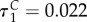, 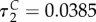, 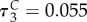 month^−1^; antibiotic clearance rate for the resistant, *a_R_* = 0 and for the sensitive *a_S_* = 30 month^−1^.

Simulations showed that equations (3.1)–(3.3) together with (3.4) are accurate (figure 1). To verify the accuracy of these expressions, we numerically computed the invasion fitness as a function of the rate of inter-class transmission. For transmission, we assumed that *β*_{_*_i_*_,*S*}→_*_j_* = *β_S_*(1 − *ɛ*) and *β*_{_*_i_*_,*R*}→_*_j_* = *β_R_*(1 − *ɛ*) for *i* = *j*; *β*_{_*_i_*_,*S*}_*_→j_* = *β_S_ɛ*/(*n* − 1) and *β*_{_*_i_*_,*R*}→_*_j_* = *β_R_ ɛ*/(*n* − 1) for *i* ≠ *j*, where *ɛ* is the proportion of inter-class transmission and *β_R_* < *β_S_* because of the cost of resistance. We varied *ɛ* from 0 (no inter-class transmission) to 1 − 1/*n* (full inter-class transmission). Treatment rate was heterogeneous across classes, but all other parameters were the same in all classes.

A small rate of inter-class transmission is sufficient to bring the system towards its expected behaviour under full inter-class transmission (figure 1). In the specific scenario for transmission rates that we considered in the simulations, the impact of inter-class transmission reduces to:
3.5
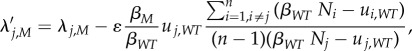
where *ɛ* is the small proportion of inter-class transmission, varying from 0 (no inter-class transmission) to 1 − 1/*n* (full inter-class transmission). If the cost of resistance is small and the difference in clearance rates across classes are small, this is approximately 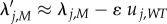: what governs the decay of invasion fitness as inter-class transmission increases is the natural clearance rate of the strain (electronic supplementary material, figure S2). In our simulations, the value of 1.1 month^−1^ typical of the species *S. pneumoniae* implies that for a resistant strain enjoying an invasion fitness of 0.05 month^−1^ (arguably a large fitness advantage, leading to a doubling time of around a year), the host population will behave as a well-mixed population as soon as *ɛ* > 0.05.

### 3.3. Analysis of the factors favouring the evolution of sensitive and resistant strains

**Figure 2.**
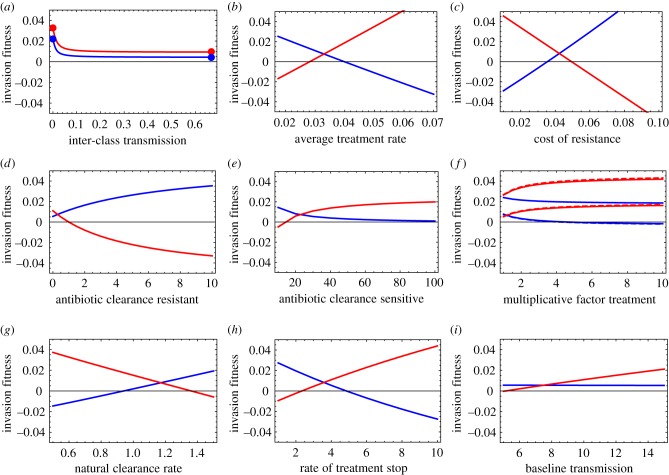
The invasion fitness *λ_S_* (blue) and *λ_R_* (red) are shown as a function of various parameters of the model. We model three classes of equal sizes *N*_1_ = *N*_2_ = *N*_3_ = 1/3. The baseline parameters are clearance rates *u_S_* = *u_R_* = 1.1 month^−1^; treatment cessation *ω* = 4 month^−1^; baseline transmission rate *β* = 10 month^−1^, inter-class transmission *ɛ* = 0.1, transmission cost of resistance *c* = 0.04; treatment varies across three classes, with rates *τ*_1_ = 0.02, *τ*_2_ = 0.035, *τ*_3_ = 0.05 month^−1^ and 10% higher in the colonized individuals, 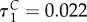, 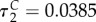, 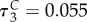 month^−1^; antibiotic clearance rate for the resistant, *a_R_* = 0 and for the sensitive *a_S_* = 30 month^−1^. In (*f*), the invasion fitness are shown for no inter-class transmission *ɛ* = 0 (top graphs) and full inter-class transmission *ɛ* = 1 − 1/*n* (bottom graphs), in order to be able to keep the fraction of treated hosts exactly constant, and the analytical results (equations (3.1) and (3.3)) are shown as dashed lines that are almost indistinguishable from the plain lines.

The expressions derived above for invasion fitness in the ‘no inter-class’ and ‘full inter-class’ transmission scenarios are accurate. The dependence of invasion fitness on parameters of the model is analogous in these two extreme scenarios of transmission, suggesting these expressions can be used to gain general analytical insights into the factors favouring antibiotic sensitivity and resistance even for more general scenarios of transmission. To do so, we computed the derivatives of the invasion fitness with respect to each parameter, for example, ∂*λ_R_*/∂*u_R_* for the clearance rate *u_R_*, and so on for the other parameters. The derivatives can be decomposed and interpreted in terms of the four phenomena that impact the strain's fitness (electronic supplementary material, table S1). We specifically computed these derivatives in the ‘full inter-class transmission’ scenario (Material and methods), and for simplicity, we presented results for the case of perfect sensitivity (*a_S_* → ∞) and perfect resistance (*a_R_* = 0). The corresponding derivatives in the ‘no transmission’ scenario can be straightforwardly obtained, and give the same insights. We complemented this analysis by investigating with simulations how invasion fitness varies as a function of each parameter (figure 2), for intermediate inter-class transmission (*ɛ* = 0.1) and imperfect sensitivity (*a_S_* = 30 month^−1^). In the following, the letters correspond to the panels in figure 2 and the derivatives are shown in electronic supplementary material, table S1.

Figure 2*a* presents the impact of inter-class transmission on the invasion fitness, as shown in figure 1. The impact of the treatment rate, the transmission cost of resistance and the antibiotic clearance rate for the resistant and sensitive strains are presented in figure 2*b–e* and readily understandable: resistance is favoured by larger rates of treatment (figure 2*b*), a smaller cost of resistance (figure 2*c*), being more perfectly resistant (figure 2*d*), and conversely for sensitivity (figure 2*b*,*c*,*e*). Other phenomena are less intuitive.

Figure 2*f*: preferentially treating the colonized rather than uncolonized hosts (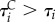), while keeping the fraction of treated host constant slightly favours the resistant strain over the sensitive strain (electronic supplementary material, table S1; figure 2*f*). Focusing the treatment on colonized individuals mainly increases clearance of the sensitive strain, and increases the number of untreated uncolonized hosts available for the resistant to colonize.

Figure 2*g*: increasing the natural clearance rates (shorter carriage duration) favours the sensitive strain [22]. For the sensitive strain, increasing the clearance rate leads to faster clearance—a detrimental effect—but also to more transmission because of an increased density of available uncolonized untreated hosts. To the leading order, the sensitive strain is favoured by increasing clearance rates thanks to its higher competitive ability (electronic supplementary material, table S1).

For the resistant strain, the opposite holds, favouring the resistant strain when natural clearance rates are smaller (longer carriage duration).

Figure 2*h*: keeping constant the fraction of hosts treated, applying more frequent but shorter-duration treatment (larger *τ_i_* and *ω_i_*) favours the resistant strain [34] because higher frequency of short treatment leads to a greater availability of untreated hosts for the resistant strain, and leads to more antibiotic clearance of the sensitive strain (electronic supplementary material, table S1).

Figure 2*i*: increasing the transmission rate enables a rare resistant strain to colonize faster the treated uncolonized hosts, increasing its invasion fitness (electronic supplementary material, table S1). The initial growth of the sensitive strains, by contrast, occurs by ecological competition with the resistant strain and displacement of the less fit resistant strain in untreated hosts, and this is little affected by the transmission rate.

### 3.4 Coexistence of *R* and *S* strains and frequency of resistance at the polymorphic equilibrium

We introduced host population structure because the rate and duration of treatment, the rate of natural clearance, the transmission rate, might vary across different host classes, and this variability across host classes will introduce variability in whether the resistant or sensitive strain is favoured in different classes, and may therefore contribute to the observed robust coexistence of sensitive and resistant strains across a wide range of average treatment rates, for example, across different countries [16].

**Figure 3.**
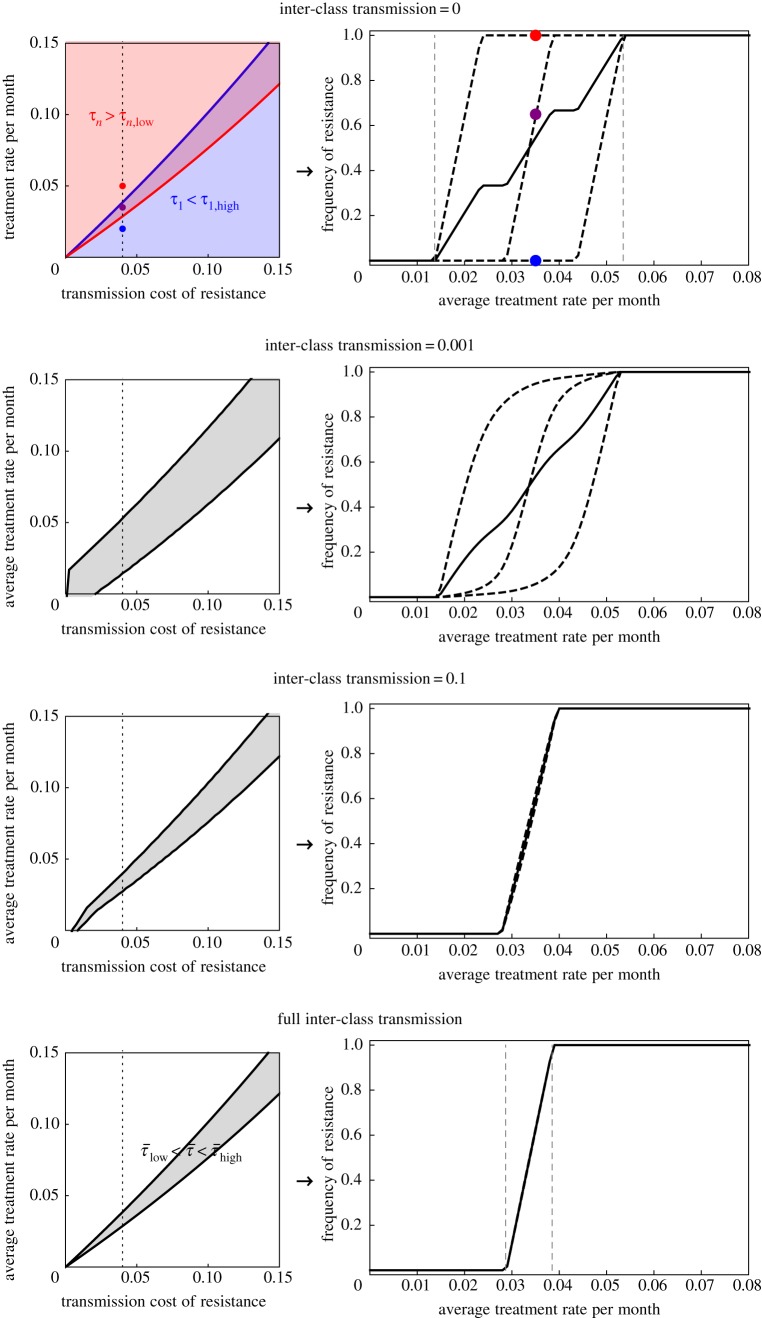
Conditions for coexistence for several levels of inter-class transmission (top to bottom). For each level of inter-class transmission, the left graph shows the condition for coexistence as a function of the cost of resistance and treatment rate. The right panel shows the global frequency of resistance (solid curve) as a function of the average treatment rate 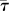, for a ‘slice’ of the left panel at cost *c* = 0.04 (marked by a dashed vertical line), for the specific example of a population subdivided in three classes with treatment rates 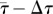, 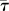 and 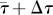 month^−1^ with Δ*τ* = 0.015. The frequency of resistance in each of the three classes (black dashed curves) is also shown. When there is no inter-class transmission (top graphs), the sensitive and resistant strains coexist if and only if *τ*_1_ is below the blue curve *τ*_1_ < *τ*_1,high_ and *τ_n_* is above the red curve *τ_n_* > *τ_n_*_,low_. The three bullets show the example population with a transmission cost of resistance *c* = 0.04 and three classes treated at rate 0.02, 0.035, 0.05 month^−1^, and where coexistence is possible because *τ*_3_ is above the red boundary and *τ*_1_ below the blue boundary. The thin purple band between the red and blue curves is where coexistence is expected if all classes used antibiotics at the same rate. In the specific example of the right panel, the limiting average treatment rates allowing coexistence are *τ*_1,low_ − Δ*τ* and *τ_n_*_,high_ + Δ*τ* (vertical grey dashed lines). Under full inter-class transmission (bottom graphs), the analytical condition for coexistence now only depends on the average treatment rate 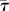, and occurs if and only if 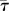 is in between the two dark lines, given by 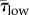 and 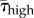 (vertical grey dashed lines on the right panel). The conditions for intermediate inter-class transmissions (*ɛ* = 0.001 and *ɛ* = 0.1, middle panels) were found by simulating the model and identifying the treatment rate at which the frequency of resistance is 0.001 (lower bound) and 0.999 (higher bound). Other parameters as in figure 1.

Coexistence occurs if and only if *λ_S_* > 0 and *λ_R_* > 0. When there is no inter-class transmission, coexistence is possible if at least one class is favourable to the sensitive strain (assumed to be class #1), and one class is favourable to the resistant strain (assumed to be class #*n*). This condition can be expressed in terms of the treatment rates in class 1 and *n*:
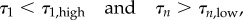
where *τ*_1,high_ is the solution of *λ*_1,*S*_ = 0 and *τ_n_*_,low_ the solution of *λ_n_*_,*R*_ = 0. Within each class, the equilibrium frequency of resistance shifts from 0 at a treatment rate *τ_i_*_,low_ to 1 at a treatment rate *τ_i_*_,high_. Global coexistence of the sensitive and resistant strains is possible, but each local class is almost always fixed for sensitivity or resistance (figure 3).

Under full inter-class transmission, when the rates of treatment cessation do not vary across classes (*ω_i_* = *ω* for all *i*), the condition can be expressed as a function of the average treatment rate experienced in the population, 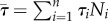, and simplifies to:
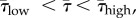
where 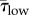 is the solution of *λ_S_* = 0 and 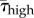 the solution of *λ_R_* = 0. This is assuming that *τ_i_* and 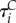 vary in the same way across classes, that is, 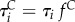 for all *i* in [1,*n*]*.* In that case, coexistence is not allowed by the heterogeneity across classes but instead by the niche formed by uncolonized untreated hosts, and the coexistence region is relatively narrow (figure 3). Differentiation across classes is sharply reduced (figure 3) and the overall frequency of resistance increases approximately linearly from 0 to 1 between 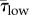 and 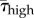.

As expected from the analysis of small rates of inter-class transmission, a small amount of inter-class transmission (*ɛ* = 0.1) is sufficient for the system to behave like a system with full inter-class transmission (figure 3).

### 3.5 Core groups of transmitters are core groups of resistance

**Figure 4.**
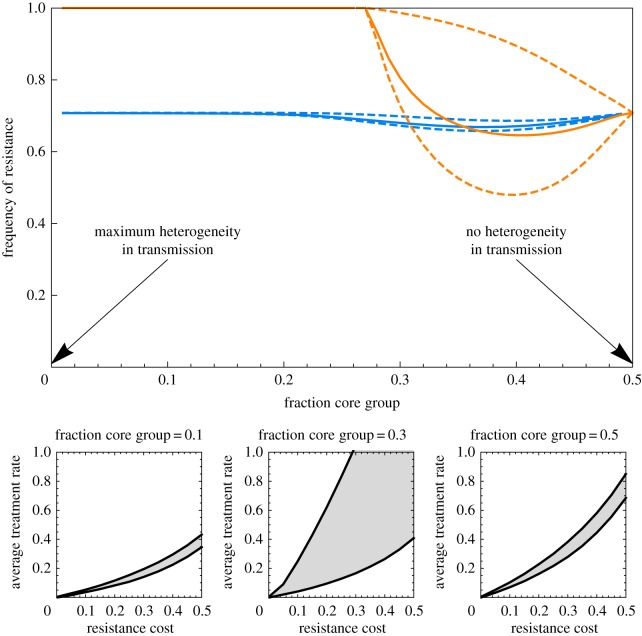
Top panel, the frequency of resistance in a host population divided into a ‘core’ and a ‘normal’ class (or group), such that the core group represents a fraction *x* of the total host density and contributes a fraction 1 − *x* of transmission, as a function of *x*. The solid line is the average frequency of resistance; the dashed lines show resistance in each of the two classes. Transmission rates were adjusted such that the reproduction number *R*_0_ of the sensitive strain in the absence of treatment stayed constant equal to 3 across values of *x*, and the core group contributed 1 − *x* to transmission. We assumed a small rate of inter-class transmission *ɛ* = 0.005. In light blue, the treatment rate is constant across classes equal to *τ*_1_ = *τ*_2_ = 0.035 month^−1^. In orange, the treatment rate is proportional to transmission, that is, 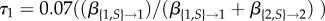 and 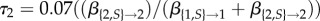 month^−1^. Bottom panel, the area of coexistence (grey) as a function of the average treatment rate and the transmission cost of resistance, when treatment rate is proportional to transmission (orange scenario in the panel above), for three chosen values of *x*. Other parameters as in figure 1.

Higher rates of transmission favour the resistant strain but not the sensitive strain (figure 2*i*, equation (3.4)). This leads us to predict that ‘core groups’ of hosts with high transmission will also be ‘core groups’ of resistance. We illustrate this finding in a scenario where the host population in subdivided into two classes: a core group (or class) with high transmission, and a low-transmission group, such that if the core group represents a fraction *x* of the total population, it contributes a fraction 1 − *x* of transmission. With *x* = 1/2, transmission is homogeneous. We let *x* vary while keeping the total basic reproduction number of the pathogen (or *R*_0_) constant. In this population, where the only difference across classes is the transmission rate, the frequency of resistance is higher in the core group (figure 4, light blue curves). If, moreover, the higher transmission rate for the focal species translates into a higher rate of all bacterial and viral infections leading to antibiotic use, the rate of antibiotic treatment will be proportional to the transmission rate, magnifying the enrichment of resistance in the core group (figure 4, orange curves). The difference in resistance between transmission groups will be substantial only when they are sufficiently isolated (in the simulations presented here, transmission within-groups is 200 more likely than transmission between-groups, *ɛ* = 0.005). Intermediate heterogeneity in transmission maximizes coexistence (figure 4): with large heterogeneity, the core group dominates and the frequency of resistance in the whole population is high, while with low heterogeneity, the population is homogeneous and little coexistence is maintained.

## 4. Discussion

We developed a model of the evolution of antibiotic resistance in a structured population, where the host population is subdivided into different classes using antibiotics at different rates. The key assumptions underlying our results are given below.

— Resistance is caused by circulating clones (primary resistance), as in many bacterial species that rarely cause infections, experience antibiotic treatment at small rates and during a short period of time, and rarely evolve de novo resistance upon treatment [35–37]. *Mycobacterium tuberculosis* (the causative agent of tuberculosis) is a counterexample: this species often causes symptomatic infections, is treated by long antibiotic courses and may evolve multiple resistant mutations upon treatment [38].
— The rate of treatment is small compared to epidemiological rates. Rates of antibiotic treatment are of the order of 1 year^−1^ in the community [16], while epidemiological rates for many bacterial species are of the order of 1 month^−1^ (e.g. [39] for *S. pneumoniae*). This assumption may not be valid for host classes such as infants who experience higher rates of treatment and/or species or strains with long carriage duration [40].
— The rates of transition between host classes are small enough to be neglected. This assumption would be valid for classes representing large geographic areas, or coarse age classes, but not, for example, finer age classes or community/hospital structure. Small rates of transition between classes are expected to have an impact similar to inter-class transmission.

Our main results concern the invasion fitness of a focal mutant strain invading a resident population at equilibrium. These analytical results give the following general insights.

(i)The resistant strain colonizes treated hosts better and is cleared less well by antibiotics, while the sensitive strain colonizes untreated hosts better. Thus, the resistant and sensitive strains rely on different strategies for replication and this ecological differentiation promotes the coexistence of these two strains in a narrow region of parameter space, even in the absence of host structure.
(ii) In a heterogeneous host population structured into different classes, low rates of inter-class transmission favour adaptation to the local conditions of each host class, and promote coexistence of sensitive and resistant strains in these different ‘niches’.
(iii) However, this phenomenon requires low inter-class transmission. In fact, we predict that the potential for pathogen local adaptation is stronger for pathogens with longer carriage duration (small rates of clearance *u_i_*_,*S*_ and *u_i_*_,*R*_, equation (3.5); electronic supplementary material, figure S2).
(iv) We examined in details the impact of epidemiological parameters on the fitness of the sensitive and resistant strains. High transmission rates favour the resistant strain, and more frequent but shorter antibiotic courses favour the resistant strain [34].
As a consequence, when transmission rates are heterogeneous, core groups of transmission have a higher frequency of resistant strains.

### 4.1. Impact of interventions to reduce antibiotic consumption

**Figure 5.**
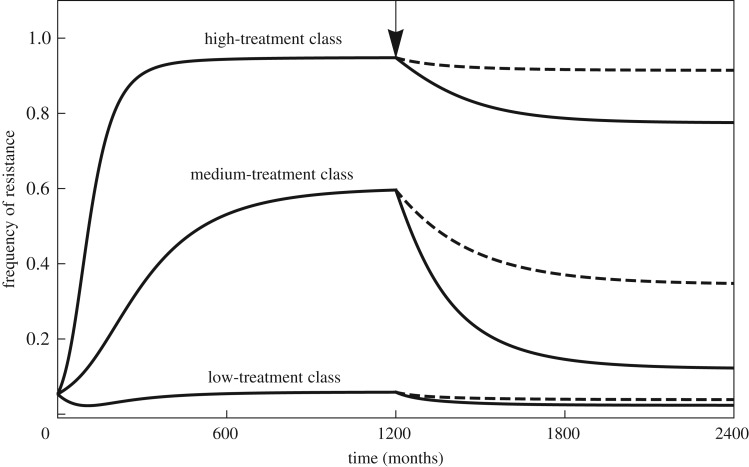
The frequency of resistance in a host population structured into three classes (bottom to top curves in order of increasing treatment rates) following an intervention at time *t* = 1200 months (shown with the arrow) to reduce the rate of antibiotic treatment (solid line) or reduce treatment duration (dashed line) by 20% in all classes. The rate of inter-class transmission is *ɛ* = 0.001. All other parameters as in figure 1.

The results can be used to investigate how levels of resistance may be reduced by public health interventions. First, reducing the treatment rate *τ_i_* is a more efficient strategy to reduce resistance than reducing the duration of the antibiotic course 1/*ω_i_* (electronic supplementary material). Reducing the treatment rate has a stronger impact on both the resistant and sensitive strains' fitness because this strategy directly reduces clearance of the sensitive strain (electronic supplementary material, table S1; figure 5).

In the context of a recent call for shorter-duration antibiotic courses [41], this result implies that reducing the duration of the course would have a smaller impact on resistance in the community than an equivalent reduction in antibiotic use. Implementing a shorter-duration course is probably easier than reducing the frequency of treatment, but it may also lead to more frequent use of antibiotics if the bacterial pathogen for which the treatment is prescribed is imperfectly cleared and a second course must be given.

**Figure 6.**
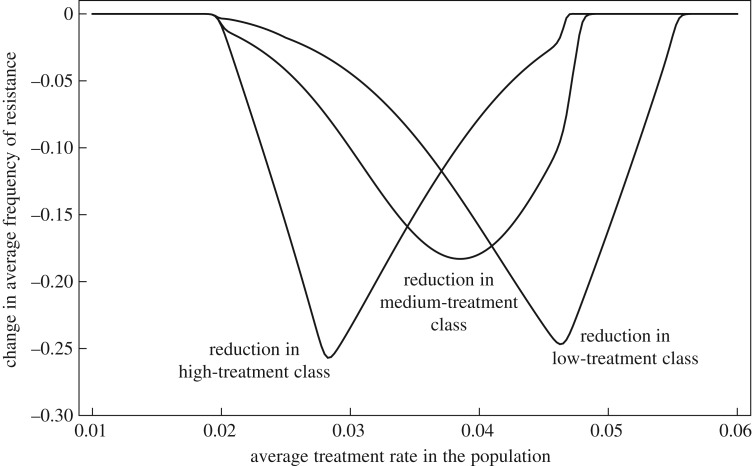
The change in the global average frequency of resistance in a host population structured in three classes following an intervention to reduce the rate of treatment by 0.01 month^−1^ in one of the classes, either the low-treatment class (right curve), the medium-treatment class (middle curve) or the high-treatment class (left curve). This is shown as a function of the global average treatment rate in the population. The rate of inter-class transmission is *ɛ* = 0.01. All other parameters as in figure 1.

Second, when host classes are sufficiently isolated, it will be more efficient to reduce resistance by reducing antibiotic use in a targeted class of hosts rather than uniformly across the host population. Determining the best class to target is difficult. Targeting a host class with a high prevalence of the bacteria will obviously have a greater impact on the overall frequency of resistance. Additionally, if the prevalence of the bacteria were the same in all classes, based on the ‘no inter-class transmission’ scenario, we hypothesize that it would be best to target a class where the planned reduction in antibiotic use can switch the class from high resistance to low resistance because selection switches from favouring the resistant to favouring the sensitive (figure 3, upper panel). This intuition is verified in simulations: it was best to target increasingly low-treatment classes when the overall average treatment rate was high (figure 6). A more thorough investigation would be required to determine how these two factors (prevalence and propensity of the class to ‘switch’) interact, and whether other phenomena complicate the picture for more general scenarios of transmission.

### 4.2. Host population structure contributes to coexistence

Coexistence between sensitive and resistant strains is favoured when parameters such as the treatment rates vary across classes of hosts. However, coexistence requires strongly isolated classes, as small rates of inter-class transmission are sufficient to mix the bacterial population well. Thus, the role of age structure [34,42,43] in maintaining coexistence is probably small, as rates of inter-age class transmission are 2–10 times lower than rates of intra-age class transmission (i.e. *ɛ* is of order approx. 0.1) [44]. Transmission rates between different countries [16] or regions [45] could be much smaller, and this type of structuration may therefore play a more important role. The epidemiological dynamics of a pathogen population imply, more generally, that pathogen genetic diversity is maintained only when the host population is strongly structured, as was also shown before in the context of the evolution of antigenic diversity [46].

## 5. Conclusion

This study develops a modelling framework to understand the evolution of antibiotic resistance in bacterial species. In future work, the major challenge will be to integrate more details of the host population and bacterial genotypes, in order to reproduce the observed pattern of resistance across settings, while retaining tractability and a good understanding of the model behaviour. This would allow more insights into the dynamics of resistance, estimating more precisely the cost of resistance, assessing the impact of a vaccine on resistance (for example, the pneumococcal conjugate vaccine against *S. pneumoniae*), and identifying the evolutionary forces selecting for multidrug resistance [47].

## Supplementary Material

Supplementary Information

## Supplementary Material

Supplementary Table

## Supplementary Material

Details of the analysis (Mathematica notebook)

## Endnote

1 For each strain, the monomorphic equilibrium is unique and a globally stable equilibrium of the single-strain subsystems as formally shown for  [33, theorem 3.2]. We conjecture this remains true in our more general model ().

## Data accessibility

This article has no additional data.
