# Supplementary material for: The evolution of antibiotic resistance in a structured host population
Source: J R Soc Interface. 2018 Jun 20;15(143):20180040. doi: 10.1098/rsif.2018.0040 (PMC6030642; doi:10.1098/rsif.2018.0040)
Supplement: Supplementary Information [file rsif20180040supp1.pdf]

Supplementary text for “The evolution of antibiotic resistance in a structured host population”

François Blanquart, Sonja Lehtinen, Marc Lipsitch, Christophe Fraser

Invasion fitness of the sensitive and resistant strains:

The outcome of the competition between a sensitive and a resistant strain depends on the invasion fitness. This is the initial exponential growth rate of a mutant when invading an equilibrium population of resident, and will be positive if the mutant can invade. Coexistence between the two strains occurs when a rare resistant strain can invade a population of sensitive at equilibrium, and reciprocally a rare sensitive strain can invade a population of resistant at equilibrium, i.e. both invasion fitness are positive. Invasion fitness is specifically the dominant eigenvalue of the matrix describing the linearized dynamics of the rare mutant. For the resistant strain, in the example of two classes of hosts (n=2), the matrix corresponding to the linearized system reads:

$$M_R = \begin{pmatrix} M_R^{U,U} & M_R^{U,T} \\ M_R^{T,U} & M_R^{T,T} \end{pmatrix}$$

where the four elements are four 2x2 matrices given by:

$$M_R^{U,U} = \begin{pmatrix} \beta_{\{1,R\}\to 1} X_1^{U*} - u_{1,R} - \tau_1^C & \beta_{\{2,R\}\to 1} X_1^{U*} \\ \beta_{\{1,R\}\to 2} X_2^{U*} & \beta_{\{2,R\}\to 2} X_2^{U*} - u_{2,R} - \tau_2^C \end{pmatrix}$$

$$M_R^{T,T} = \begin{pmatrix} \beta_{\{1,R\}\to 1} X_1^{T*} - u_{1,R} - a_R - \omega_1 & \beta_{\{2,R\}\to 1} X_1^{T*} \\ \beta_{\{1,R\}\to 2} X_2^{T*} & \beta_{\{2,R\}\to 2} X_2^{T*} - u_{2,R} - a_R - \omega_2 \end{pmatrix}$$

$$M_R^{U,T} = \begin{pmatrix} \beta_{\{1,R\}\to 1} X_1^{U*} + \omega_1 & \beta_{\{2,R\}\to 1} X_1^{U*} \\ \beta_{\{1,R\}\to 2} X_2^{U*} & \beta_{\{2,R\}\to 2} X_2^{U*} + \omega_2 \end{pmatrix}$$

$$M_R^{T,U} = \begin{pmatrix} \beta_{\{1,R\}\to 1} X_1^{T*} + \tau_1^C & \beta_{\{2,R\}\to 1} X_1^{T*} \\ \beta_{\{1,R\}\to 2} X_2^{T*} & \beta_{\{2,R\}\to 2} X_2^{T*} + \tau_2^C \end{pmatrix}$$

where X_1^{U*} and X_2^{U*} are the equilibrium density of uncolonised, untreated hosts when the sensitive strain (resident) is at equilibrium in the population, and X_1^{T*} and X_2^{T*} are the equilibrium densities of uncolonised, *treated* hosts when the sensitive strain is at equilibrium in the population. The matrix describing initial growth of the sensitive strain, M_S, is analogous. Coexistence occurs if and only if $\lambda_S > 0$ and $\lambda_R > 0$, where λ_S and λ_R are the dominant eigenvalues of the matrices M_S and M_R. We first derive an expression for the eigenvalues as a function of the equilibrium density of uncolonised X_i^{U*} and X_i^{T*} when the resistant strain is alone (for λ_S) and when the sensitive strain is alone (for λ_R). Then we derive expressions for these equilibria (X_i^{U*} and X_i^{T*}) to finally obtain closed-form expressions for λ_S and λ_R. Calculations are detailed in a companion Mathematica notebook.

Equilibrium expressions for the densities of uncolonised hosts at the resident strain equilibrium

Equation (3) in the main text can be completed with the equilibrium expressions for the densities of resident uncolonised hosts at equilibrium. These are:

$$X_j^{U*} = \frac{u_{j,WT}}{\beta_{\{j,WT\}\to j}} + \frac{\Gamma_{j,WT}}{\beta_{\{j,WT\}\to j}}$$

$$X_j^{T*} = \frac{\mathrm{E}_{j,WT}}{\beta_{\{j,WT\}\to j}}$$

$$X_j^{U*\prime} = X_j^{U*} - \frac{u_{j,WT}}{\beta_{\{j,WT\}\to j}} \sum_{\substack{i=1 \\ i\neq j}}^{n} \left(\frac{\beta_{\{i,WT\}\to j}}{\beta_{\{j,WT\}\to j}}\right) \left(\frac{N_i - u_{i,WT}/\beta_{\{i,WT\}\to i}}{N_j - u_{j,WT}/\beta_{\{j,WT\}\to j}}\right)$$

$$X^{U*} = \frac{u_{WT}}{\beta_{WT}} + \frac{1}{\beta_{WT}} \sum_{i=1}^{n} N_i\, \Gamma_{i,WT}$$

$$X_i^{T*} = \frac{\mathrm{E}_{i,WT}}{\beta_{WT}}$$

and to the first order, the ratios X_i^{U*}/X^{U*} appearing in equation (3) in the antibiotic clearance term are simply approximated as N_i. The subscript “WT” denotes the resident wild type. The density of uncolonised untreated hosts depends on

$$\Gamma_{j,WT} = \frac{\tau_j^C\left(a_{WT}\,\omega_j - u_{j,WT}\left(N_j\,\beta_{\{j,WT\}\to j} - u_{j,WT}\right)\right) - \tau_j\, u_{j,WT}(\omega_j + u_{j,WT})}{\omega_j\left(a_{WT} + N_j\,\beta_{\{j,WT\}\to j} + \omega_j\right)},$$

a small term representing the impact of antibiotic treatment. The density of uncolonised treated hosts depends on

$$E_{j,WT} = \frac{\tau_j\, u_{j,WT}\,(u_{j,WT} + \omega_j + a_{WT}) + \tau_j^C (u_{j,WT} + a_{WT})(N_j \beta_{\{j,WT\}\to j} - u_{j,WT})}{\omega_j (N_j \beta_{\{j,WT\}\to j} + \omega_j + a_{WT})},$$

a small positive term.

Impact of reducing treatment rate or reducing treatment duration on resistance

When both resistant and sensitive strains coexist in the population, the equilibrium frequency of resistance is well predicted by the ratio of invasion fitnesses of the resistant and the sensitive strain (sup. fig. 1). To investigate how a change in treatment rates (τ_i and τ_i^C) or treatment duration (ω_i) would affect the frequency of resistance, therefore, we study how a change in these parameters impact the invasion fitnesses. We do so in the 'full inter-class transmission' scenario and we expect results to be similar in the 'no inter-class transmission' scenario.

From the derivatives in Supplementary Table 1, a small change $\Delta\tau$ (negative for a reduction in treatment) in the treatment rate applied uniformly across host classes, would result in the following change in invasion fitnesses:

$$\Delta\lambda_R = \frac{\Delta\tau}{\tau}\left(\beta_R\, T^{S*} + \frac{\beta_R}{\beta_S}\tau^{\mathrm{C}}\right)$$

$$\Delta\lambda_S = -\frac{\Delta\tau}{\tau}\left(u_R\frac{\beta_S}{\beta_R}\left(T^{R*} - \frac{\beta_R - u_R}{\beta_R}\sum_{i=1}^{n}\frac{N_i(\tau_i^C - \tau_i)}{\beta_R + \omega_i}\right) + \tau^C\right)$$

where $T^{S*} = \sum_{i=1}^{n} N_i \frac{\tau_i^C}{\omega_i} - \frac{u_S}{\beta_S}\sum_{i=1}^{n} N_i \frac{\tau_i^C - \tau_i}{\omega_i}$ is the fraction of hosts under treatment in the population at equilibrium with the sensitive strain only and similarly, $T^{R*} = \sum_{i=1}^{n} N_i \frac{\tau_i^C}{\omega_i} - \frac{u_R}{\beta_R}\sum_{i=1}^{n} N_i \frac{\tau_i^C - \tau_i}{\omega_i}$ is the equilibrium fraction of hosts under treatment in the population at equilibrium with the resistant strain only.

From the derivatives in Supplementary Table 1, a small change $\Delta\omega$ (assumed to be positive) in the rate of treatment cessation would result in the following change in invasion fitness:

$$\Delta\lambda_R = \frac{\Delta\omega}{\omega}\beta_R\, T^{S*}$$

$$\Delta\lambda_S = \frac{\Delta\omega}{\omega}u_R\frac{\beta_S}{\beta_R}\left(T^{R*} - \frac{\beta_R - u_R}{\beta_R}\sum_{i=1}^{\mathrm{n}}\frac{N_i(\tau_i^C - \tau_i)}{\beta_R + \omega_i}\frac{\omega_i}{\beta_R + \omega_i}\right)$$

In these equations, the terms $\Delta\tau/\tau$ (resp. $\Delta\omega/\omega$) represent the magnitude of the intervention, and the remainder of the equation represents the impact of the intervention. Reducing the

treatment rate directly reduces the resistant strain fitness and increases the sensitive strain fitness (term in τ^C), an effect that reducing treatment duration does not have. Thus, the impact of reducing the treatment rate on resistance is always greater than the impact of reducing treatment duration, $\beta_R\, T^{S*} + \frac{\beta_R}{\beta_S}\tau^{\mathrm{C}} > \beta_R\, T^{S*}$. The beneficial impact of reducing the treatment rate on the sensitive strain is also always greater than the impact of reducing treatment duration when $\tau_i^C = \tau_i$, and numerical investigation suggest this is also likely to be true even when $\tau_i^C > \tau_i$ for plausible parameter values.

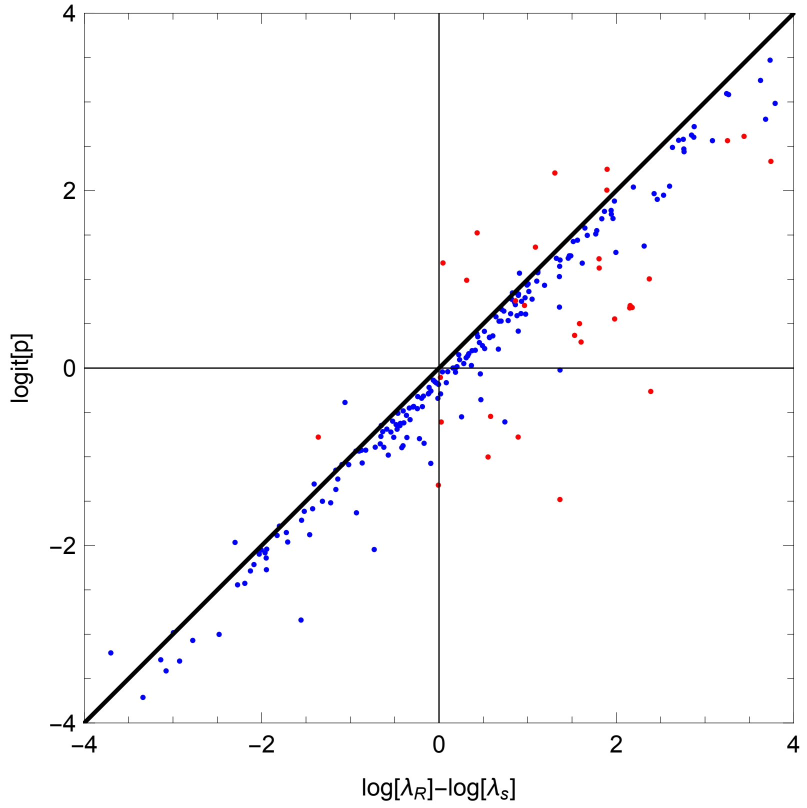

Supplementary Figure 1: the frequency of resistance correlates well with the invasion fitnesses. logit[p] is shown as a function of $\log[\lambda_R] - \log[\lambda_S]$ across 228 random simulations where coexistence between the sensitive and the resistant strain occurred. Parameters are: three classes of size N_i drawn independently in each class in a uniform[0,1] then rescaled to $N_1 + N_2 + N_3 = 1$. Clearance rates $u_{i,S} = u_{i,R}$ drawn independently in each class in a uniform[0.5, 2] month^{-1}; treatment rates τ_i drawn independently in each class in a uniform[0,0.1] month^{-1} and $\tau_i^C = f^C\,\tau_i$ with the factor f^C drawn in a uniform[1, 1.5]; treatment cessation ω_i drawn independently in each class in a uniform[3, 5]; baseline transmission rate β drawn in a uniform[2, 8]; inter-class transmission ε drawn in a uniform [0, 1-1/n]. The blue points show the simulations with high inter-class transmission (values of $\varepsilon \geq 0.01$) and the red points show the values for low inter-class transmission ($\varepsilon < 0.01$). Cost of resistance c drawn in a uniform [0, 0.05]; antibiotic clearance rate for the resistant, $a_R = 0$ and for the sensitive $a_S = 30$ month^{-1}.

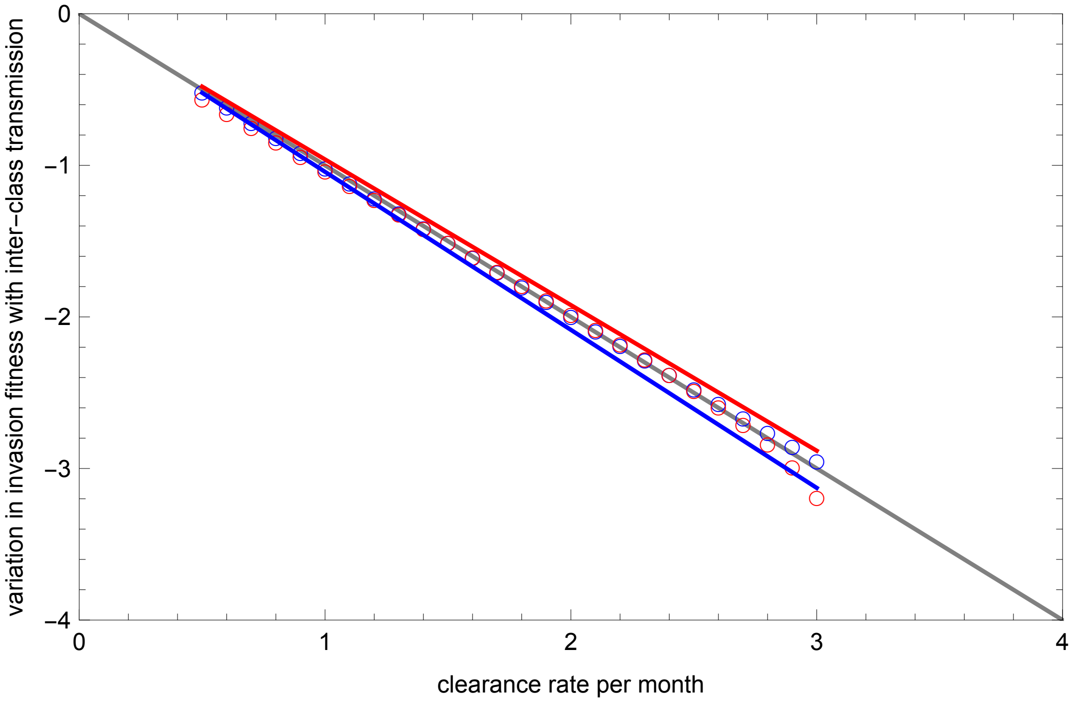

Supplementary Figure 2: the decay of invasion fitness for the sensitive (blue) and the resistant (red) strains primarily depend on the clearance rate per month. We varied the clearance rate from 0.5 to 3 per month to simulate different strains with different epidemiological turnover rates. We computed the initial rate of decay of invasion fitness $\Delta\lambda/\varepsilon$ when a little inter-class transmission $\varepsilon = 0.001$ was added. The points are the simulations, the lines the analytical expressions (equation (5) in the main text). For transmission, we assumed that $\beta_{\{i,S\}\to j} = \beta_S(1-\varepsilon)$ and $\beta_{\{i,R\}\to j} = \beta_R(1-\varepsilon)$ for $i = j$; $\beta_{\{i,S\}\to j} = \beta\ \varepsilon/(n-1)$ and $\beta_{\{i,R\}\to j} = \beta_R\ \varepsilon/(n-1)$ for $i \neq j$, where ϵ is the proportion of inter-class transmission and $\beta_R < \beta_S$ because of the cost of resistance. Other parameters as on fig. 1.
