# Supplementary material for: The evolution of antibiotic resistance in a structured host population
Source: J R Soc Interface. 2018 Jun 20;15(143):20180040. doi: 10.1098/rsif.2018.0040 (PMC6030642; doi:10.1098/rsif.2018.0040)
Supplement: Supplementary Table [file rsif20180040supp2.pdf]

Supplementary Table 1 for "The evolution of antibiotic resistance in a structured host population" - François Blanquart, Sonja Lehtinen, Marc Lipsitch, Christophe Fraser

List of the derivatives showing the impact of each parameter on the sensitive and resistant invasion fitness. Each derivative was decomposed into the sum of four terms (four columns) for better interpretation. The first column refers to the corresponding figure 2 panel (B to I). For simplicity, they are shown for a perfectly sensitive and resistant strain, $a_S \to \infty$ and $a_R = 0$, except for the derivatives with respect to a_S and a_R.

The derivatives with respect to the treatment rate (**B**, $\partial\lambda_S/\partial\tau$ and $\partial\lambda_R/\partial\tau$) are calculated assuming all rates τ_i and τ_i^C are proportional to the same variable, and taking the derivative with respect to this variable.

The derivatives with respect to the treatment rate in colonised over that in uncolonised hosts (**F**, $\partial\lambda_S/\partial f^C$ and $\partial\lambda_R/\partial f^C$) are calculated keeping the fraction of treated hosts constant. For example, in a population where only the sensitive strain is present, in the full transmission scenario, the fraction of host under treatment is $\sum_{i=1}^{n} N_i \frac{\tau_i^C}{\omega_i} - \frac{u_S}{\beta_S}\sum_{i=1}^{n} N_i \frac{\tau_i^C - \tau_i}{\omega_i}$. Thus, to keep the fraction of treated host constant across values of f^C, we suppose that the ratio τ_i^C/τ_i is constant across classes, that is $\tau_i^C = \tau_i\, f^C$, and we rescale $\tau_i \sim \frac{\beta_S}{f^C\beta_S - u_S\, f^C + u_S}\,\tau_i$ and $\tau_i^C \sim f^C \frac{\beta_S}{f^C\beta_S - u_S\, f^C + u_S}\tau_i$ (the scaling is $\tau_i \sim \tau_i$ when $f^C = 1$). A similar approach can be used for $\partial\lambda_S/\partial f^C$, and in the no inter-class transmission scenario. In the no inter-class transmission scenario, when only the sensitive bacteria is present, the fraction of treated hosts in class n is $\frac{\tau_n^C}{\omega_n} - \frac{u_{n,S}}{N_n\, \beta_{\{n,S\}\to n}}\frac{\tau_n^C - \tau_n}{\omega_n}$. Thus, we rescale $\tau_n \sim \frac{\beta_{\{n,S\}\to n}\, N_n}{f^C\beta_{\{n,S\}\to n}\, N_n - u_{n,S}\, f^C + u_{n,S}}\,\tau_n$ and $\tau_n^C \sim f^C \frac{\beta_{\{n,S\}\to n}\, N_n}{f^C\beta_{\{n,S\}\to n}\, N_n - u_{n,S}\, f^C + u_{n,S}}\tau_n$.

The derivatives $\partial\lambda_S/\partial\omega$ and $\partial\lambda_R/\partial\omega$ are first calculated directly (line 3 and 4 in the table below). Then, they are calculated while keeping the fraction of treated hosts constant (**H**). To do so, we assume ω_i, τ_i and τ_i^C are all proportional to the same variable, and we calculate the derivative with respect to that variable.

Fig. 2 panel	Derivative	Natural clearance	Colonisation of untreated	Colonisation of treated	Antibiotic clearance
B	$\partial\lambda_S/\partial\tau$	0	$-\frac{\beta_S}{\beta_R}\frac{u_R}{\tau}\sum_{i=1}^{n} N_i \frac{\tau_i(u_R+\omega_i)+\tau_i^C(\beta_R-u_R)}{\omega_i(\beta_R+\omega_i)}$	0	$-\frac{\tau^C}{\tau}$
B	$\partial\lambda_R/\partial\tau$	0	$\frac{\beta_R}{\beta_S}\frac{\tau^C}{\tau}$	$\frac{\beta_R}{\beta_S}\sum_{i=1}^{n} N_i \frac{\tau_i\, u_S+\tau_i^C(\beta_S-u_S)}{\omega_i\, \tau}$	0
	$\partial\lambda_S/\partial\omega$	0	$\frac{\beta_S}{\beta_R}\frac{u_R}{\omega}\sum_{i=1}^{n} N_i \frac{\tau_i(u_R\beta_R+2u_R\omega_i+\omega_i^2)+\tau_i^C(\beta_R-u_R)(\beta_R+2\omega_i)}{\omega_i(\beta_R+\omega_i)^2}$	0	0
	$\partial\lambda_R/\partial\omega$	0	0	$-\frac{\beta_R}{\beta_S}\sum_{i=1}^{n} N_i \frac{\tau_i\, u_S+\tau_i^C(\beta_S-u_S)}{\omega_i\, \omega}$	0
C	$\partial\lambda_S/\partial c$	0	$\frac{u_R}{(1-c)^2}\left(1+O(\tau)\right)$*	0	0
C	$\partial\lambda_R/\partial c$	0	$-u_S-\tau^C$	$-\sum_{i=1}^{\mathrm{n}} N_i \frac{u_S\,\tau_i+(\beta_S-u_S)\,\tau_i^C}{\omega_i}$	0
D	$\partial\lambda_S/\partial a_R$	0	$\frac{\beta_S}{\beta_R}\sum_{i=1}^{n} N_i \frac{(u_R+\omega_i)\left(\tau_i\, u_R+\tau_i^C(\beta_R-u_R+\omega_i)\right)}{\omega_i(a_R+\beta_R+\omega_i)^2}$	$\frac{\beta_S(\beta_R-u_R)}{\beta_R}\sum_{i=1}^{\mathrm{n}} N_i \frac{u_R\,\beta_S+\beta_R\,\omega_i}{u_R\,\beta_S+\beta_R(a_S+\omega_i)}\frac{\tau_i\, u_R+\tau_i^C(\beta_R-u_R+\omega_i)}{\omega_i(a_R+\beta_R+\omega_i)^2}$	0
D	$\partial\lambda_R/\partial a_R$	0	0	$-\beta_R\sum_{i=1}^{n} N_i \frac{u_S\,\beta_R+\beta_S\,\omega_i}{(u_S\,\beta_R+\beta_S(a_R+\omega_i))^2}\frac{\tau_i\, u_S(a_S+u_S+\omega_i)+\tau_i^C(a_S+u_S)(\beta_S-u_S)}{\omega_i(a_S+\beta_S+\omega_i)}$	$-\beta_S\sum_{i=1}^{n} N_i\, \tau_i^C \frac{u_S\beta_R+\beta_S\,\omega_i}{\left(u_S\beta_R+\beta_S(a_R+\omega_i)\right)^2}$

E	$\partial\lambda_S/\partial a_S$	0	0	$-\beta_S\sum_{i=1}^{n} N_i \frac{u_R\beta_S+\beta_R\,\omega_i}{\left(u_R\,\beta_S+\beta_R(a_S+\omega_i)\right)^2}\frac{\tau_i\,u_R(a_R+u_R+\omega_i)+\tau_i^C(a_R+u_R)(\beta_R-u_R)}{\omega_i(a_R+\beta_R+\omega_i)}$	$-\beta_R\sum_{i=1}^{n} N_i\,\tau_i^C \frac{u_R\,\beta_S+\beta_R\,\omega_i}{(u_R\,\beta_S+\beta_R\,(a_S+\omega_i))^2}$
E	$\partial\lambda_R/\partial a_S$	0	$\frac{\beta_R}{\beta_S}\sum_{i=1}^{n} N_i \frac{(u_S+\omega_i)(\tau_i\,u_S+\tau_i^C(\beta_S-u_S+\omega_i))}{\omega_i(a_S+\beta_S+\omega_i)^2}$	$\frac{\beta_R(\beta_S-u_S)}{\beta_S}\sum_{i=1}^{n} N_i \frac{u_S\beta_R+\beta_S\,\omega_i}{u_S\beta_R+\beta_S(a_R+\omega_i)}\frac{\tau_i\,u_S+\tau_i^C(\beta_S-u_S+\omega_i)}{\omega_i(a_S+\beta_S+\omega_i)^2}$	0
F	$\partial\lambda_S/\partial f^C$	0	$\frac{\beta_S}{\beta_R}\frac{u_R(\beta_R-u_R)}{u_R+f^C(\beta_R-u_R)}\sum_{i=1}^{n}\frac{N_i\tau_i}{\beta_R+\omega_i}$	0	$-\frac{u_R\,\tau}{u_R+f^C(\beta_R-u_R)}$
F	$\partial\lambda_R/\partial f^C$	0	$\frac{\beta_R}{\beta_S}\frac{u_S}{u_S+f^C\,(\beta_S-u_S)}\sum_{i=1}^{n} N_i\,\tau_i$	0	0
G	$\partial\lambda_S/\partial u$	-1	$\frac{\beta_S}{\beta_R}\frac{u_R}{u_S}\left(1-\sum_{i=1}^{n} N_i \frac{\tau_i(2u_R+\omega_i)+\tau_i^C(\beta_R-2u_R)}{\omega_i(\beta_R+\omega_i)}\right)$	0	0
G	$\partial\lambda_R/\partial u$	$-\frac{u_R}{u_S}$	$\frac{\beta_R}{\beta_S}$	$-\frac{\beta_R}{\beta_S}\sum_{i=1}^{n}\frac{N_i(\tau_i^C-\tau_i)}{\omega_i}$	0
H	$\partial\lambda_S/\partial\omega^{**}$	0	$\frac{\beta_S}{\beta_R}\frac{u_R(\beta_R-u_R)}{\tau}\sum_{i=1}^{n} N_i \frac{\tau_i^C-\tau_i}{(\beta_R+\omega_i)^2}$	0	$-\frac{\tau^C}{\tau}$
H	$\partial\lambda_R/\partial\omega^{**}$	0	$\frac{\beta_R}{\beta_S}\frac{\tau^C}{\tau}$	0	0
I	$\partial\lambda_S/\partial\beta$	0	$-u_R\sum_{i=1}^{n} N_i \frac{(\tau_i^C-\tau_i)(u_R+\omega_i)}{\omega_i(\beta_R+\omega_i)^2}$	0	0

I	$\partial\lambda_R/\partial\beta$	0	0	$\frac{\beta_R}{\beta_S}\sum_{i=1}^{n}\frac{N_i\,\tau_i^C}{\omega_i}$	0

* $O(\tau)$ denotes a small complicated term of the same order as the treatment rates τ_i and τ_i^C.

** in that case, the derivatives with respect to ω were calculated while keeping the fraction of treated hosts constant.
